# Method for selective quantification of immune and inflammatory cells in the cornea using flow cytometry

**DOI:** 10.14440/jbm.2018.237

**Published:** 2018-11-22

**Authors:** Mamoru Ogawa, Takenori Inomata, Tina Shiang, Kazuo Tsubota, Akira Murakami

**Affiliations:** 1 Laboratory for Metabolomics, RIKEN Center for Integrative Medical Sciences, Tsurumi-ku, Yokohama 230-0045, Japan; 2 Department of Ophthalmology, Keio University School of Medicine, Tokyo 160-8582, Japan; 3 Department of Ophthalmology, Juntendo University Faculty of Medicine, Tokyo, 113-0033, Japan; 4 Department of Strategic Operating Room Management and Improvement, Juntendo University Faculty of Medicine, Tokyo, 113-0033, Japan; 5 University of Massachusetts Medical School, Department of Radiology, MA 01655, USA

**Keywords:** cornea, flow cytometry, angiogenesis, inflammation, immune privilege, transplantation, wound healing

## Abstract

The cornea serves as a protective surface against the environment (*i.e.*, allergens, pollutants, desiccation and microorganisms) and promotes vision, made possible by corneal transparency. This protocol describes corneal preparation for flow cytometry to assess cells localized in the cornea. Our model details the process, from determining how many corneas are needed in the experiment to corneal excision to digestion and staining of the cornea cells. The simplicity of the model allows for systematic analysis of different corneal mechanisms of immunity, inflammation, angiogenesis and wound healing. In corneal transplantation, residential immune and inflammatory cells are key to the mechanisms that underlie angiogenesis, opacity, and graft rejection. In addition, this model can also elucidate cellular mechanisms mediating corneal graft outcomes and wound healing. Lastly, this model can be used to analyze the efficacy of new medications such as instillation and subconjunctival injections and assess the potential of therapeutic molecules to enhance graft survival and wound healing *in vivo*.

## BACKGROUND

The ocular surface is continuously exposed to environmental agents such as allergens, pollutants, desiccation and microorganisms [1]. The smooth wet surface of the cornea is the major refractive surface of the visual system [2], made possible by corneal transparency. When this environment collapses due to infection or inflammation, the normally transparent and avascular cornea develops opacification and neovascularization [3,4], resulting in decreased visual acuity. Dry eye disease induces ocular damage by the inflammatory cascade from innate and adaptive immune responses [5-7]. In the corneal wound healing process, opacity occurring due to corneal stromal fibroblasts and myofibroblasts is problematic [8]. Corneal transplantation is performed to address the corneal opacity. However, high-risk recipient corneas with angiogenesis experience acute rejection in greater than 40%–90% of cases [3,9].

The cornea can be observed optometrically and is an excellent choice for analyzing the efficacy of new medications such as instillation and subconjunctival injection [10,11]. Clinical and experimental studies are investigating the use of hyaluronic acid and fibronectin for wound healing [12-14], steroids and immunosuppressants [15,16], and even anti-VEGF drugs for neovascularization, inflammation and rejection [17-20]. In wound healing, immune cells migrate along neovessels in the transparent corneal tissue [6,21,22], so controlling the corneal environment is important. Immunostaining, PCR, and western blotting can be used to confirm immune cells and inflammatory cells localized in the cornea, but flow cytometry is the most effective method of quantification of inflammatory cells in the cornea.

Much research on corneal flow cytometry has been done so far [9,23]. Previously, studies have shown that immune cells circulate in the cornea and cervical lymph nodes to control the immunocompetence of the cornea [24]. Furthermore, immune cells localized in the cornea affect rejection, angiogenesis, and wound healing [8,25]. However, since the murine cornea is small and it is difficult to obtain cells abundantly, it is important to understand how to obtain accurate results from experiments.

Therefore, this paper introduces a flow cytometry protocol for murine corneas. Briefly, we introduce the process from cornea harvesting to digestion and flow cytometry preparation. Furthermore, we delineate the number of corneas needed for successful flow cytometry and provide steps for troubleshooting.

## MATERIALS

### Reagents

Corneas from age- and sex-matched mice were used to decrease variability. Depending on the aim of the study, this technique can be applied to different strains, genders, ages and animal models.

- DNase I (Roche, Basel, Switzerland, cat. # 10104159001)
- Collagenase D (Sigma-Aldrich, St. Louis, MO, cat. # 11088882001)
- RPMI-1640 media (Lonza, Walkersville, MD, cat. # 12-702F)
- Fetal Bovine Serum (ATLANTA biological, GA, cat. # S11550)
- Phosphate-Buffered Saline (PBS) (CORNINIG, MA, cat. # 21-0404-CMR)
- Bovine Serum Albumin (BSA) (SIGMA-ALDRICH, MO, cat. # A2153-100G)
- Fc receptor blocking antibody (eBioscience, San Diego, CA, cat. # 14-0161-85)
- PE-anti-mouse CD45 Antibody (BioLegend, San Diego, CA, cat. # 103106)
- FITC anti-mouse CD4 Antibody (BioLegend, CA, cat. # 100510)
- Propidium iodide solution (Sigma-Aldrich, MO, cat. # P4864)

### Equipment

- Scissors (Vannas and curved)
- 1.5 ml Sampling Tubes (WATSON, cat. # 131-415C)
- 12 × 75 mm Polystyrene Culture Tube (FACS tube) (QSP, CA, cat. # 530-B-1Q)
- Sterile Jeweler’s forceps (one blunt and one fine)
- 6 well Multidish (IWAKI, Japan, cat. # 3810-006)
- 70 μm cell strainer (FALCON, USA, cat. # 352350)
- 22 G 5 ml Syringe (TERUMO, Japan, cat. # ss-05Sz2232)
- Sterile needle 30 G (M-S Surgical, Japan, cat. # 12745002)
- Binocular microscope
- Flow cytometer (BD FACScantoTM П Flow Cytometer)
- FlowJo software X 10.0.7 (FlowJo LLC, Ashland, OR, USA)

## PROCEDURE

All animal experiments conducted in this protocol were approved by the Institutional Animal Care and Use Committee of the Juntendo University Faculty of Medicine (cat. # 290120) and RIKEN Center for Integrative Medical Sciences (cat. # 29-009-4).

### Cornea harvesting

**Figure 1.**
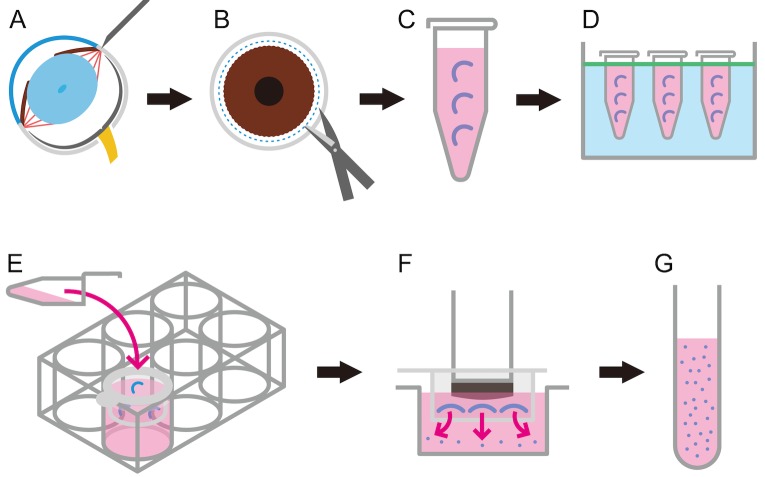
Schematic drawing of the harvesting and digestion of corneal tissue for flow cytometry. **A.** Corneal perforation at the limbal area using a 30 G needle. **B.** Cut the cornea by Vannas scissors. **C.** Preserve tubes with DNase І and collagenase D. **D.** Incubate in a 37°C bath. **E.** Transfer to a 70 μm mesh on a 6 well plate with RPMI. **F.** Mash cells using a syringe. **G.** Transfer cells to the tube for flow cytometry.

***1.*** Punch out the corneal limbus with a 30 G needle (**Fig. 1A**). In parallel to the iris, punch outside the limbus and reduce the sclera as much as possible.
***2.*** Cut the cornea circularly from the puncture wound at the corneal limbus with Vannas scissors (**Fig. 1B**).
***3.*** Peel the iris from the back of the cornea under a microscope.

### Cornea digesting

***4.*** Add DNase І (0.002 g) and collagenase D (0.004 g) in 1 ml of RPMI-1640 to a 1.5 ml tube (ideally up to 4 corneas per tube) (**Fig. 1C**).
***5.*** Incubate solution in a 37°C bath for 60 min (**Fig. 1D**), inverting every 10 min (while pipetting up and down).
***6.*** Add 200 μl of FBS to the tube to stop the DNase І and collagenase D, and add the tube contents to a cell strainer in a 6-well plate with 5 ml RPMI for filtering (**Fig. 1E**).

### Single cell suspension

***7.*** Mash the corneas using the plunger of a 5 ml syringe (**Fig. 1F**).
***8.*** Add filtrate to the FACS tube (**Fig. 1G**). Wash the filter and dish with 5 ml RPMI-1640 and transfer to the FACS tube.

### Flow cytometry

***9.*** Centrifuge the FACS tube at 1300 RPM for 10 min. Pour and discard the supernatant.
***10.*** Add 2 ml of FACS buffer (1% BSA with PBS) and centrifuge at 1300 RPM for 10 min. Pour and discard the supernatant.
***11.*** Add 1 μl of Fc receptor blocking antibody to each sample and keep the samples at 4°C on the shaker for 30 min.
***12.*** Add the surface antibodies (*i.e.*, CD45 and CD4) directly to each sample, and keep the samples at 4°C for 1–2 h.
***13.*** Add 2 ml of FACS buffer to each sample and centrifuge at 1300 RPM for 10 min. Pour and discard the supernatant.
***14.*** Add 300 μl of FACS buffer with propidium iodide solution (0.5 mg/ml) and run flow cytometry.
***15.*** Results were analyzed using FlowJo software.
***16.*** Doublets discrimination was performed by forward scatter (FSC) and side scatter (SSC) gating.
***17.*** Dead cells should be excluded from further analysis.

## ANTICIPATED RESULTS

**Figure 2.**
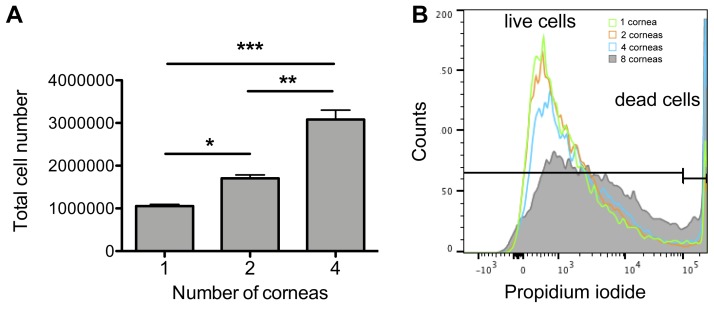
Anticipated total number of cornea cells and leukocytes by flow cytometry. **A.** The figure shows the anticipated number of cornea cells obtained from C57BL6 mice using this protocol. **B.** The live/dead cells in the total number of cornea cells and leukocytes. *P* values are calculated using one-way ANOVA with Bonferroni post hoc test, and error bars represent SEM. *P < 0.05, **P < 0.01 and ***P < 0.001.

**Figure 2A** shows the total number of cornea cells and passenger leukocytes obtained, counted using binocular microscope. When using between one and four corneas, the number of single suspended cells can be collected as expected. However, loss of single suspended cells was noted at eight corneas (See Troubleshooting). The frequencies of live/ dead cells were shown in **Figure 2B**. Among the cells obtained after doublets discrimination, over 90% were live cells between one and four corneas, however the dead cells were increased at eight corneas. Therefore, the ideal number of corneas to be used with the amount of lysis reagent in our protocol is four corneas. Based on this result, it is possible to set up an experiment plan corresponding to the required number of cells.

Our model allows for detailed analysis of immune pathways of interest, including the Th1 immune response, regulatory effect of T cells, pro-inflammatory and tolerogenic capacity of antigen presenting cells, and the role of innate and adaptive immunity in angiogenesis [9,23]. We also recommend this protocol for assessing the changes in cornea cells in the wound healing process. Moreover, this model can be used with knockout and transgenic mice to evaluate various molecular pathways participating in the immune response, angiogenesis and wound healing processes.

**Figure 3.**
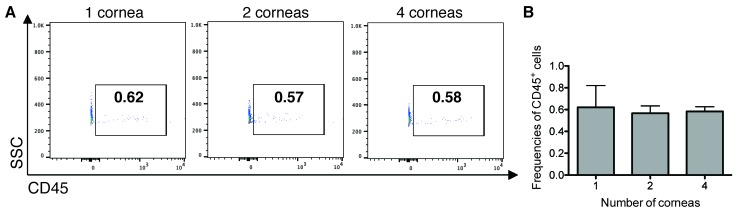
CD45^+^ leukocytes cornea cells. **A.** and **B.** Representative flow cytometry dot plots (A) and bar graphs showing frequencies of CD45^+^ leukocytes among corneal cells from naïve BALB/c mice (B). Bar graphs show mean ± SEM.

Other models for assessing cornea cells have been used previously and may complement the model proposed in this paper. Real-time PCR and western blot analysis can be used for quantification of corneal cells, but it is difficult to accurately measure the limited amount of RNA and protein in individual corneas, and the aforementioned methods are not effective for residential cells that require multiple staining. **Figure 3** shows CD45^+^ leukocytes in corneal cells induced by inflammation [23,26]. Our protocol enables the detection of dynamic trends of localized cells in the cornea during wound healing.

**Figure 4.**
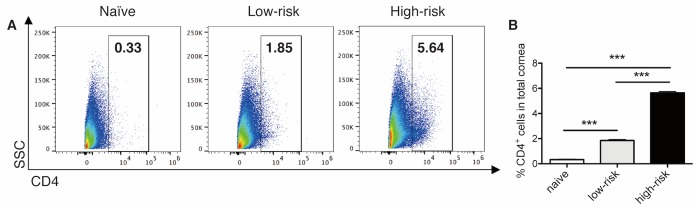
CD4^+^ T cells 14 days post-transplantation. **A.** and **B.** Representative flow cytometry dot plots (A) and bar graphs showing the frequencies of CD4^+^ T cells of corneal cells among naive cornea, low-risk corneal transplantation and high-risk corneal transplantation (B). *N* = 5 mice/group, data represents results of three independent experiments. *P* values are calculated using one-way ANOVA with Bonferroni *post hoc* test, and error bars represent SEM. *P < 0.05, **P < 0.01 and ***P < 0.001.

In this model, you can detect dynamic changes in local corneal cells used in conjunction with other models such as the eye drop challenge, medical injections to the eye, wound healing and corneal transplantation model. Previously, we published the protocol for the high-risk corneal transplantation model [27]. Briefly, inflamed and neovascularized (high-risk) host beds (BALB/c) were created by three intrastromal sutures placed into the central cornea using 11-0 nylon sutures (AB-0550S, MANI, Tochigi, Japan) 14 days before corneal transplantation to induce host beds prone to reject the grafts. Mice with unmanipulated (low-risk) host beds served as controls. For allogeneic corneal transplantation, C57BL/6 corneas were grafted onto BALB/c host beds. **Figure 4** shows CD4^+^ T cells in corneal cells post-transplantation. We can detect recruited CD4^+^ T cells post-transplantation and demonstrate significantly increased CD4^+^ T cells in high-risk inflamed corneal transplantations compared to low-risk corneal transplantations.

Overall, the simplicity, availability, and the cost-effectiveness of the described model provide a unique platform to study basic principles of immunity and angiogenesis for ophthalmic pathologies.

## TROUBLESHOOTING

Here we list potential issues and provide troubleshooting instructions.

**Figure 5.**
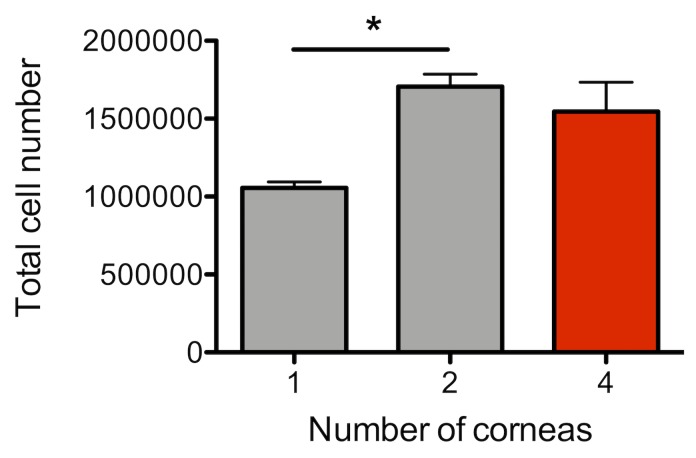
Cell loss in single cell suspension. The number of cells obtained when the cell suspension was performed with only 1 ml of RPMI for filtering step. *P* values are calculated using one-way ANOVA with Bonferroni *post hoc* test, and error bars represent SEM (*P < 0.05).

If few cells can be collected, melting time may be too short or the mesh time for the single cell suspension may be insufficient. Consider increasing the dissolution time and mesh time. **Figure 5** shows the number of cells obtained when the cell suspension was performed with only 1 ml of RPMI. If the RPMI is not sufficient for the single cell suspension, cells may remain in the mesh. Cell loss may occur, a problem that worsens as the number of corneas increases. Therefore, we recommend preparing 5 ml or more of RPMI when doing a single cell suspension in a 6 well plate.

**Figure 6.**
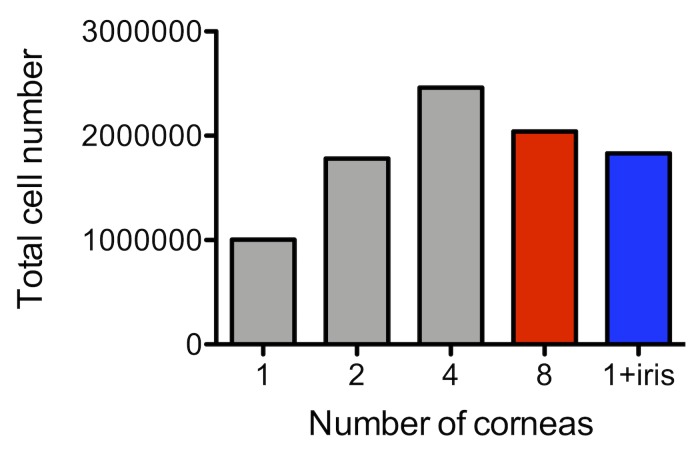
Cell loss due to too many corneas and cell increase due to iris. If there are too many corneas, the cell suspension cannot be performed sufficiently resulting in cell loss. If the iris is not removed completely, the number of cells will increase.

If single cells cannot be isolated due to too many cornea samples, adjust the amount of DNase І and collagenase D as necessary. **Figure 6** shows that the number of cells increases with the number of corneas up to four corneas. However, beyond four corneas, cell loss occurs.

Since the number of cells is falsely increased if iris samples are included (**Fig. 6**), peel off the iris as much as possible when collecting the cornea.

Possible problems and their troubleshooting solutions are listed in **Table 1**.

## Figures and Tables

**Table 1.** Troubleshooting for single cell suspension.

Problem	Cause	Suggestion
Few cells	Short melting time or mesh time Lack of RPMI-1640	Increasing the dissolution time or mesh time Enough RPMI-1640 (5 ml or more RPMI-1640)
Cell loss	Too many cornea samples	Adjust the amount of DNase І and collagenase D (Procedure 4)
Increased cells	Contamination of other tissues	Peel off the iris as much as possible when collecting the cornea
